# Health literacy and ethnic disparities in health-related quality of life among rural women: results from a Chinese poor minority area

**DOI:** 10.1186/1477-7525-11-153

**Published:** 2013-09-11

**Authors:** Cuili Wang, Hui Li, Lingui Li, Dongjuan Xu, Robert L Kane, Qingyue Meng

**Affiliations:** 1 Shandong University School of Nursing, 250012 Jinan, China; 2 Shandong University Center for Health Management and Policy, 250012 Jinan, China; 3 Ningxia Medical University College of Management, 750004 Yinchuan, China; 4 University of Minnesota School of Public Health, 55455 Minneapolis, US; 5 Peking University China Center for Health Development Studies, 100191 Beijing, China

**Keywords:** Health-related quality of life, EQ-5D, Health literacy, Race/ethnicity, Equity, China

## Abstract

**Background:**

We examined the relationship between health literacy (HL) and health-related quality of life (HRQoL) as well as relationship differentials by ethnicity among rural women from a Chinese poor minority area.

**Methods:**

We conducted in-person interviews with 913 rural women aged 23 – 57 (57.5% Hui minorities/42.5% Han ethnicity) enrolled in the Ningxia Women Health Project, gathering data on EQ-5D, self-designed HL, socio-demographic characteristics, and chronic diseases. The extent of impairments in the five dimensions of the EQ-5D was used to measure HRQoL. Factor analysis yielded a single HL factor, which was used as a dichotomous variable in multivariate log-binomial regression models that examined the adjusted association of HL with HRQoL.

**Results:**

Nearly half of the women had no formal education. The most prevalent impairments were pain/discomfort and anxiety/depression (42.42% and 32.09%, respectively). The Hui minorities had 1.65 times higher rates of low HL (defined as less than mean of the factor score for HL) and 1.22 and 1.25 times for pain/discomfort and anxiety/depression impairments, respectively. Low HL was associated with poor HRQoL, with a 23% increase in the prevalence of pain/discomfort impairments after adjusting for socio-demographics. This association was significant in the Hui group (PR=1.30, 95% CI=1.06-1.58) but not for the Han group (PR=0.99, 95% CI=0.76-1.30). HL-stratified analysis revealed modification for ethnic disparities in HRQoL; for pain/discomfort impairments, high HL-PR=0.88 (95% CI=0.71-1.08), low HL-PR=1.24 (95% CI = 1.01-1.52); for anxiety/depression impairments, high HL-PR=0.98 (95% CI=0.73-1.32), low HL-PR=1.44 (95% CI = 1.05-1.98).

**Conclusions:**

Low HL is associated with poor HRQoL across the entire sample and the association may be modified by ethnicity. Similarly, ethnic disparities in HRQoL may be modified by HL, larger in low HL group. Health services should address HL in vulnerable minority women to improve their HRQoL.

## Background

Health-related quality of life (HRQoL), representing people’s subjective assessment of their sense of well-being and ability to perform physical, psychological and social functions, has been increasingly used as a comprehensive health indicator in medical interventions or health surveys [1,2]. HRQoL is viewed as a primary patient-centered outcome for care. Minorities typically have lower HRQoL. This disparity has widened in recent decades, despite an increase in the overall level of health status [3,4]. Eliminating these disparities has become a universal priority. However, progress has been slow and our understanding of the underlying causes of health disparities remains limited [4].

HL plays a pivotal role in public health and medical settings. Low HL is prevalent among racial/ethnic minorities [5-7]. Low HL is consistently associated with poor health outcomes, such as global health status, mortality, hospitalization and preventive health care use [7], but data on its relationship with HRQoL are insufficient and equivocal [5,7-11]. For example, HL showed a significant association with mental health dimension as measured by SF-12 among U.S. elders [10] but not among Korean elders [9]. Another study using EQ-5D to measure HRQoL showed no significant relationship between HL and HRQoL among patients with rheumatic diseases [12]. However, despite these mixed findings, theoretical models provide three potential pathways that link low HL to health disparities: 1) decreased accessibility and use of medical care because individuals with low HL may have difficulty navigating the health care system, 2) increased stress burden by increasing the challenges of daily life and health system navigation and illness self-management, and 3) decreased self-efficacy, i.e. ‘the ability to exert control over one’s life and surroundings’ [4,13]. In addition, racial/ethnic disparities in HRQoL (beyond socioeconomic status) were reduced when HL was considered [10,14]. Therefore, we believe that enhancing HL could reduce health disparities.

Low HL may interact with racial/ethnic minority status to affect health status [15], but few studies tested this hypothesis. Sudore and colleagues found that the interaction between race and limited literacy on mortality in the elderly was not significant and the relationships between limited literacy and high mortality were consistent across African American and Caucasian subgroups [13]. However, one study of low-income female caregivers showed that low oral HL was weakly associated with more oral HRQoL impacts across the entire sample, and the association was more pronounced among African Americans than that among Caucasians [16]. Most program and policy analyses rely on the simple evaluation of the impacts of HL in the general population. Using such evaluation underestimates and even masks the variation within racial/ethnic groups. Further insight is needed into the relationship between HL and HRQoL among more diverse community-based samples. Additionally, because HL is a multifaceted and comprehensive concept, reading fluency should not be the sole indicator measured.

Although HL research has proliferated during the past two decades, unified definitions and measures are still lacking. The definitions proposed by the American Medical Association (AMA) [17], the Institute of Medicine (IOM) [18] and the World Health Organization (WHO) [19] are most often cited. These share an emphasis on individual capacities to access, understand, and use health information and services to make appropriate health decisions [20]. However, the constituent dimensions of HL remain disputed. IOM views health knowledge as part of HL in addition to the basic components of many definitions, which include oral and print literacy and numeracy [18]. Lee et al. identify four interrelated factors of disease and self-care knowledge, health risk behavior, preventive care and physician visits, and compliance with medication [21]. Nutbeam distinguishes three levels of HL: basic/functional, interactive/communicative, and critical [22]. HL measurement instruments have limitations, as well. Most measure only word recognition or reading comprehension and numeracy and overlook all other components of HL, such as oral skills and critical thinking, in part because it is difficult to assess such a vast body of core essentials in medical settings [6,20,23,24].

More comprehensive definitions of HL have been suggested. Singleton proposed a working definition of HL in the Virginia Adult Education HL Toolkit: “HL is the knowledge and skills needed to be aware of one’s own health beliefs and practices” [25]. WHO also highlighted HL as a key outcome from health education [19]. Recently Sorensen et al. on behalf of the European Health Literacy Project (HLS-EU) proposed an integrative definition based on a systematic review [20]. That is, HL refers to the knowledge, motivation and competencies of accessing, understanding, appraising and applying health-related information within the healthcare, disease prevention and health promotion setting to maintain or improve quality of life during the life course [20]. We use this as our operational definition, arguing that HL is the comprehensive competency to process health information and services to make judgments and decisions in daily life. HL is made up of health-related knowledge, presenting health information skills, health beliefs, and health behaviors.

In China, HL is a new concept and mainly employed in public health rather than clinical settings. In 2008, Chinese Ministry of Health developed a measure “HL for Chinese Citizens--Basic Knowledge and Skills”. This 66-item questionnaire addresses three components: basic knowledge and belief, health lifestyle and behaviors, and basic skills [26]. Using this questionnaire, a nationally representative survey of 79,542 people showed that only 6.48% of residents have adequate HL; there were urban–rural and regional differentials, with lower percentage of adequate HL in rural and western residents compared to their counterparts [27]. The concept of HL is sensitive to context and culture, and its meaning can differ across cultures/countries or racial/ethnic groups [4,23]. Some Chinese researchers attempted to develop measurement instruments embedded in Chinese culture. Li Xiao et al. screened indicators of HL by Delphi method and constructed a comprehensive index of HL and four sub-indexes of health knowledge, health behavior, health belief and health skills [28,29]. Nevertheless, Chinese instruments have measured cognition and application of health information without testing reading and numeracy skills. Many studies have focused solely on the prevalence and distribution of HL in a population; however, few have examined the relationship between HL and health outcomes, or the interaction between low HL and social vulnerabilities.

Clarification is needed regarding the relationship of HL with HRQoL among a diverse racial/ethnic sample. This would provide important information from which to more accurately assess the effects of interventions on subpopulations. Such information is urgently needed for several reasons: first, the importance of HRQoL and HL measures and their inconsistent relationship both in medical care and population settings; second, the paucity of information on racial/ethnic differentials in their association; and third, the growing imperative in eliminating health disparities.

The Ningxia Women Health Project, implemented in Guyuan, Ningxia Hui Autonomous Region, aimed to improve the health status of a vulnerable population of rural women in Northwestern China. Using the survey data, we examined the association between HL and HRQoL among rural women in this region. We also explored any difference in this association between ethnic groups (the Han majorities and the Hui minorities).

## Methods

### Study setting and sampling

Our study is based on a field survey in June 2010 with cross-sectional data from Guyuan City, a prefecture-level city of Ningxia Hui Autonomous Region. Ningxia is an underdeveloped region in Northwestern China. Guyuan is located at the northwest edge of the Loess Plateau and in the Liupanshan mountain area of Southern Ningxia. In 1972, the United Nations declared this region unsuitable for human survival. Guyuan is the poorest area in China; the disposable income per capita of rural residents was 2962 yuan (434US$) in 2009 compared to 5153 yuan (754US$) for the whole country. The population of Guyuan was about 1.5 million, 45.3% of which were Hui minorities who practice the Islam faith. The education level of the region, with an average of six years of schooling among rural and urban women aged 18 and older, is far lower than the national level and eastern level.

The study was approved by the Institutional Review Board from Shandong University. Our survey included Guyuan’s one and only district (Yuanzhou) and two of its four counties (Xiji and Pengyang). Seven elementary schools were randomly selected from the two counties and one district. Trained investigators received referrals for families of all students from schools and 913 mothers participated in the study, yielding a response rate of 81.16%. Subjects participated in an in-person structured interview, and provided written consent regarding our collection and use of the data. Interviewees in this study were informed about the purposes and objectives of this study and agreed to participate.

### Measurements

#### *Health-related quality of life (HRQoL)*

Health-related quality of life was measured using EQ-5D (European Quality of Life-5 Dimensions), consisting of five dimensions (mobility, self-care, usual activities, pain/discomfort and anxiety/depression). EQ-5D has been used in the National Health Survey in China and is well validated [30]. Participant responses indicate three levels of severity (no problems/ moderate problems/extreme problems) within a particular EQ-5D dimension. We reported them as dichotomous variables (no impairment versus any impairment).

#### *Socio-demographic characteristics*

Participants’ self-reported ethnicities were classified as either Han ethnicity or the Hui minorities. Age in years, educational level and household economic status and a geographical variable were included. The highest attained educational level was classified as no formal schooling, elementary school (grade 1 – 5), middle school (grade 6 – 8) or higher. Household economic status was measured by a dichotomous variable (poverty or non-poverty), where the poor criterion was household per capita disposable income in 2009 less than the median of 2962 yuan (434US $). Geographical location was trichotomized into the mountain area, the plain area, and the edge of mountain and plain area; the mountain area is the harshest natural and living environment, more deficient in good soil and water resources, and poorer in infrastructure.

#### *Chronic diseases*

Respondents were asked a simple dichotomous question: “Have you been diagnosed with chronic diseases during the past 6 months? (yes/no). ”

#### *Health literacy*

Efforts have been made to achieve a sufficiently comprehensive measure of HL [26,28,29,31]. However, the HL measures in the study were only a small part of the survey and thus did not allow for a long HL questionnaire. Based on the operational definition and KAB (knowledge-attitude-behavior) theory extensively used in health education and our review of other comprehensive instruments, we selected some commonly used indicators and adapted them to the lower educational level of surveyed women. Table 1 shows these indicators in relation to dimensions of HL. Health knowledge indicators involved knowledge that women used in daily life such as risk factors of chronic diseases, prevention and treatment strategies for nutritional and communicable diseases, and the effect of mental problems. Health information presenting skill addressed the complexity of reading comprehension and numeracy in medical settings, as measured by answering the contraindication, identifying the expiry date, and calculating the dose after reading a medication instruction. To measure health belief, we used such indicators as the views on health congenital attributes, the weighted value of money and health, and regular physical examination. Health behavior involved regular and patterned behaviors related to health including health-seeking behaviors and medication management. For the indicators of the first three dimensions, the indicators were valued as “1” if respondents answered correctly, otherwise “0”. For the last dimension, as respondents were asked to report frequency of acquiring health information, the indicator was valued as “1” for “often or sometimes” and as “0” for “seldom or never”. Keeping common medicines at home was valued as “1” and not doing so “0”. Seeking a doctor or taking medicine by oneself was valued as “1” and delay or no treatment as “0.” Content Validity Indexes (CVIs) were computed to provide content validity of the scale [32], using ratings of item relevance by 6 experts from health education field. The item-level CVIs (I-CVIs) for each item ranged from 0.83 to 1 and scale-level CVI (S-CVI) for the total scale was 0.91, which suggests the scale has excellent content validity in terms of Polit’s criteria [32].

**Table 1:** HL dimensions and related indicators

**Dimensions**	**Indicators**
**Health knowledge**	**Please answer the following questions:**
	Is high-salt diet associated with hypertension?
	Is obesity associated with hypertension?
	Can calcium deficiency be prevented by exposing them to more sunshine?
	Must rabies vaccine be injected after one is bitten by domesticated cats or dogs?
	Can long-term depressed mood or high stress affect physical health?
**Health information presenting skill**	**Please look through the instruction for the medicine and answer the following questions:**
	Is this medicine out the date or expired?
	How much is the adult dose of the medicine a day?
	Can a child aged one and a half years old take this medicine?
	How many packages should a child weighting 30 kilograms take a day?
**Health belief**	**Do you agree with the following views?**
	Health is natural or genetic phenomenon and no one can change it.
	I care more about earning a lot of money than about health.
	Only people aged 50 and older people need care for their blood pressure.
	Healthy people don’t need to have regular physical examination.
**Health behavior**	**What do you do in your life?**
	Do you take initiative to access health knowledge?
	Do you keep some common medicines at home?
	What actions do you usually take when you are ill or feel discomfort?

### Statistical analyses

The statistical analyses included two stages: (1) Factor analysis was used to extract a HL factor; (2) Log-binomial regression models were used to explore the relationship between HL, ethnicity, and HRQoL, which could produce the prevalence ratios (PRs) more interpretable and correct for cross-sectional studies with common outcomes, as opposed to odds ratios (ORs) in logistic regression models which will overestimate the risk ratio when it is more than 1 [33,34]. All data were analyzed with the STATA 12.0.

### Factor analysis

The means of the four dimensions of HL are used in an exploratory factor analysis of the principal components. A single factor was extracted with eigenvalues above 1.0 that accounted for 54.27% of the total variance (Table 2). Factor loadings were all above 0.4; they met the psychometric criteria and the factor was named HL (Table 2). The HL factor score was calculated by a regression equation, which may accurately reflect the level of HL with an inclusion of the weighting factors against dimensions. Its internal consistency reliability was relatively good (Cronbach’s α=0.713).

**Table 2:** **Factor analysis**^**1 **^**of HL**

**Variables**	**Factor loading**	**Factor scoring coefficients**
Health knowledge	0.73	0.34
Health information presenting skill	0.76	0.35
Health belief	0.78	0.36
Health behavior	0.68	0.31
Eigenvalue	2.17	
% of variance	54.27	

**1:** Factor analysis method: principal-component factors.

Factor score method: regression.

This HL instrument was developed based on revision of the Chinese Adult Health Literacy Questionnaire (CAHLQ) [29] with incorporation of direct measure of reading and numeracy skills in the U.S. [24], so norms or thresholds for what constitutes “low HL” have not been established. HL has been classified as high and low HL in terms of the criterion of the median score for a revised HL scale based on the Test of Functional Health Literacy in Adults (TOFHLA) [9]. Additionally, nearly half of study participants had no formal education, suggesting a potentially very low level of HL since HL is strongly associated with education [7]. Therefore, the mean of the factor score was used to determine low or high HL in the study. The HL factor score was dichotomized into a binary variable: high HL (factor score ≥ 0) and low HL (factor score < 0). It was used into the following log-binomial regression models.

### Log-binomial regression models

Hierarchical log-binomial regression models were fit for each dimension of the EQ-5D to examine the main effect of HL and ethnicity and the potential mediation of HL on ethnic disparities in HRQoL. Groups of variables were sequentially entered: socio-demographics (age, ethnicity, education, income, and geographical location), HL, and chronic diseases. PRs with 95% confidence intervals (CIs) were calculated on the binary response (EQ-5D impairments) by changes in the predicting variable. Next, we assessed potential effect modification by ethnicity. To identify how HL was associated with HRQoL within ethnic groups, we conducted ethnicity-stratified analyses and ran high and low HL subgroup models to determine the different extent of ethnic disparities in HRQoL. The statistically significance of interaction terms between ethnicity and HL was assessed by Wald χ^2^ tests.

## Results

### Characteristics of respondents

Table 3 shows the characteristics of the sample. Respondents’ average age was 35 years; nearly half were Han and had no formal schooling. Women in the Hui minority group were younger, nearly twice as likely to have had no formal schooling, more likely to live in mountain area, and more likely to have low HL and chronic diseases. The pain/discomfort dimension had the highest prevalence for all rural women (42.42%), followed by anxiety/depression (32.09%). A higher percentage of Hui minorities reported impairments in the above two dimensions of EQ-5D (P < 0.05) than those for the Han ethnicity. The prevalence in other three dimensions of EQ-5D was low (from 5.59% through 14.80%) and had no significant ethnic difference.

**Table 3:** Sample characteristics and the prevalence of EQ-5D impairments by ethnic subgroups

**Variables**	**Total sample**	**Ethnic subgroups**		**P**^**1**^
		**Han**	**Hui**	
	**(n=913)**			
		**(n=388)**	**(n=525)**	
**Age**, mean (SD)	35.18 (4.71)	36.12 (4.40)	34.49 (4.82)	0.000
**Hui minority** (%)	57.50			
**Education** (%)				
no formal schooling	47.21	30.93	59.24	0.000
Elementary school	28.37	36.08	22.67	
Middle school or more	24.42	32.99	18.10	
**Income**: poverty (%)	48.56	47.01	49.71	0.422
**Geographical location** (%)				0.000
Mountain area	30.11	22.60	35.62	
Plain area	51.43	57.14	47.24	
The edge of mountain and plain	18.46	20.26	17.14	
**Presence of chronic disease** (%)	38.16	29.64	44.47	0.000
**Low HL** (%)	50.66	36.65	60.58	0.000
**EQ-5D: any impairment** (%)				
Mobility	8.65	7.47	9.52	0.276
Self-care	5.59	5.15	5.90	0.626
Usual activities	14.80	16.28	13.71	0.281
Pain/discomfort	42.42	37.63	45.98	0.012
Anxiety/depression	32.09	27.98	35.11	0.023

1: t test was used to compare the difference of women’s age; χ^2^ tests were used to compare the differences of percentages for categorical variables between ethnic subgroups.

### The relationship between HL and HRQoL

Table 4 presents the PRs and 95% CI for ethnicity and HL on EQ-5D dimensions for the total sample. The PRs, relative to the high HL group show somewhat increased prevalence in HRQoL impairments for the low HL group. For example, with adjustment for socio-demographics (Model 2), women with low HL had significantly higher prevalence of pain/discomfort impairments (PR=1.23, 95% CI=1.01–1.50). However, this association became non-significant with further adjustment for chronic diseases (Model 3). Similarly, significant socio-demographic-adjusted ethnic disparities in prevalence of pain/discomfort and anxiety/depression impairments (Model 1), with higher relative risk among Hui minorities, slightly declined and persisted with an addition of HL (Model 2). However, the disparities were eliminated with further adjustment for chronic diseases. Additionally, the presence of chronic diseases was significantly associated with any impairment in all EQ-5D dimensions, with the prevalence ratios ranging from 2.14 through 4.07 (not shown).

**Table 4:** **Adjusted**^**1 **^**prevalence ratios (PRs) and 95% CI for EQ-5D impairments for total sample**

**EQ-5D impairments**	**Model 1**	**Model 2**	**Model 3**
	**PRs (95% CI)**	**PRs (95% CI)**	**PRs (95% CI)**
**Mobility**			
Hui (ref. Han)	1.19 (0.76–1.87)	1.12 (0.71–1.77)	0.95 (0.61–1.47)
Low HL (ref. high HL)		1.74 (0.97–3.13)	1.67 (0.94–2.98)
**Self-care**			
Hui (ref. Han)	1.18 (0.66–2.10)	1.2 (0.67–2.15)	1.01 (0.57–1.79)
Low HL (ref. high HL)		0.84 (0.42–1.69)	0.81 (0.41–1.59)
**Usual activities**			
Hui (ref. Han)	0.90 (0.65–1.26)	0.90 (0.65–1.26)	0.80 (0.58–1.11)
Low HL (ref. high HL)		1.0 (0.66–1.51)	0.96 (0.64–1.43)
**Pain/discomfort**			
Hui (ref. Han)	1.23(1.04–1.46)	1.21 (1.02–1.44)	1.02 (0.89–1.18)
Low HL (ref. high HL)		1.23 (1.01–1.50)	1.13 (0.95–1.33)
**Anxiety/depression**			
Hui (ref. Han)	1.31 (1.06–1.63)	1.31 (1.05–1.62)	1.17 (0.95–1.44)
Low HL (ref. high HL)		1.07 (0.83–1.38)	1.04 (0.82–1.32)

1: Log-binomial regression models. Model 1 introduced socio-demographics (age, ethnicity, education, income, geographic location). Model 2 further introduced HL based on model 1. Model 3 further introduced the presence of chronic diseases based on model 2.

Table 5 presents adjusted PRs and 95% CI for EQ-5D impairments by ethnicity and HL, respectively. The ethnicity-stratified analyses showed that the significant association of low HL with more pain/discomfort impairments persisted for the Hui subgroup (PR=1.30, 95% CI=1.06–1.58), even with further adjustment for chronic diseases, but not for the Han subgroup (PR=0.99, 95% CI=0.76–1.30). Similar patterns of associations between HL and anxiety/depression impairments were found between the Hui and Han groups, although their association was not significant for either group. The HL-stratified analyses showed that Hui minorities had a significantly increased relative risk of pain/discomfort impairments, compared to the Han women among the low HL group (PR=1.24, 95% CI=1.01–1.52), but a decreased relative risk among the high HL group (PR=0.88, 95% CI=0.71–1.08). Similar HL patterns of ethnic disparities in anxiety/depression impairments were found: low HL-PR=1.44 (95% CI=1.05–1.98); high HL-PR=0.98 (95% CI=0.73–1.32). The interaction term of ethnicity with HL in the fully adjusted model was statistically significant for anxiety/depression impairments (P=0.046), suggesting that ethnicity may modify the effect of HL, and that HL may modify ethnic disparities in HRQoL, as well.

**Table 5:** **Adjusted**^**1 **^**prevalence ratios (PRs) and 95% CI for EQ-5D impairments, by ethnicity and HL categories**

**Model**	**Ethnicity subsamples**		**HL subsamples**		**P**^**2**^
	**Han**	**Hui**	**High HL**	**Low HL**	
	**Low HL (ref. High HL)**	**Low HL (ref. High HL)**	**Hui (ref. Han)**	**Hui (ref. Han)**	
**EQ-5D**	PRs (95% CI)	PRs (95% CI)	PRs (95% CI)	PRs (95% CI)	
Mobility	1.54 (0.65–3.63)	1.77 (0.79–3.99)	0.59 (0.25–1.39)	1.10 (0.65–1.87)	0.709
Self-care	0.79 (0.26–2.36)	0.80 (0.33–1.91)	1.03 (0.43–2.46)	1.11 (0.51–2.42)	0.870
Usual activities	0.76 (0.44–1.32)	1.19 (0.66–2.12)	0.81 (0.50–1.30)	0.89 (0.56–1.40)	0.526
Pain/discomfort	0.99 (0.76–1.30)	1.30 (1.06–1.58)	0.88 (0.71–1.08)	1.24 (1.01–1.52)	0.099
Anxiety/depression	0.86 (0.57–1.30)	1.17 (0.86–1.58)	0.98 (0.73–1.32)	1.44 (1.05–1.98)	0.046

1: Log-binomial regression models, adjusting for age, income, education, geographical location and the presence of chronic diseases.

2: P for interaction by wald χ2 test between ethnicity and HL.

## Discussion

The HRQoL among rural women in this harsh, economically depressed area was very poor. Compared to the Chinese national female representative sample [30] as well as rural residents in Western China [35], the respondents reported higher percentages of impairments in all EQ-5D dimensions. Pain/discomfort and anxiety/depression were the impairments that most strongly affected Chinese rural women’s HRQoL. Consistent with studies in China and other countries [30,35-37], the prevalence of mobility, self-care and usual activities impairments was quite low. Female characteristics in the study may make these findings more pronounced because previous studies showed that women reported more impairments in EQ-5D dimensions than men, especially in pain/discomfort and anxiety/depression dimensions [30,36,38,39]. Further, the participants were young and physically healthy women, so a ceiling effect may contribute to lower prevalence of mobility, self-care and usual activities across Han and Hui women based on the strong negative association of HRQoL and physical health with age [30]. Nevertheless, women here experienced higher impairments in the above three dimensions of EQ-5D compared to the national sample. This suggests that minority women have greater need for effective measures to improve their HRQoL.

Our study found that women with low HL had a higher risk of pain/discomfort impairments across the entire sample. After adjusting for socio-demographics, there was a negative association between HL and HRQoL. Different population and contexts may contribute to the inconsistency of the association shown in previous studies. HL may exert stronger influences on HRQoL when it is below a certain threshold [16]. Ethnic disparities in pain/discomfort and anxiety/depression impairments persisted with inclusion of HL but were eliminated with inclusion of chronic diseases. This suggests that higher prevalence of chronic diseases, rather than low HL among minorities, may be a strong attribution or mediator [1].

The association of HL with pain/discomfort impairments varied by ethnicity; low HL appeared to be harmful for the Hui minorities but not for the Han group. Our findings suggest that vulnerabilities such as limited HL often coexist and interact with other social vulnerabilities [15]. Low HL may exacerbate ethnic disparities in HRQoL among rural women. The results of both the present study and previous studies emphasize that the presence of chronic diseases account for poorer HRQoL [1,40,41]. Low HL is significantly associated with having chronic diseases [42]. Additionally, ethnic disparities in chronic diseases persist, with our study showing 1.5 times higher prevalence of chronic diseases among Hui minorities compared to the Han group. Given these established associations of chronic diseases-strong risk factor for HRQoL impairments-with HL and ethnicity, we expected that low HL would be associated with HRQoL impairments, and that the association might even be stronger in Hui minorities compared to the Han group.

Additionally, the fact that HRQoL estimates are essentially reporting of health-related problems and subjective health status and women with lower HL were less expressive of HRQoL impacts associated with diseases compared to those with higher HL [41] could explain the inverse association between HL and HRQoL impacts among the Han group. Ethnic disparities in their association can be attributed to the poorer social sources among Hui minorities as well. The two ethnic groups differed in education levels, as well. The percentage of illiterate women among Hui minorities was nearly twice that for the Han group. Many Hui women were deprived of education rights during their childhood because of such feudal ideas as “male superiority to female”. Lack of formal education prevented these women from gaining the basic knowledge and skills necessary to access information and resources and to initiate behaviors that enhance health [43], all factors that in turn lower HL level. Residential segregation may also play a role in ethnic disparities in health [44]. Hui minorities were more likely to reside in poor mountain areas with very little arable land and poor access to and quality of health care. As a result, many of these rural women live in extreme poverty and even fall sick from heavy farm burden [44].

The data did not permit us to investigate psychological and cultural mechanisms of ethnic disparities in the association between HL and HRQoL. However, past research on racial/ethnic disparities suggests that chronic stress associated with social disadvantages was the proximate cause of health disparities along with access to medical care, environmental exposure, and health behavior [4,43,44]. Therefore, higher perceived anxiety/depression among the Hui minority women may reflect the elevated stress response. Moreover, some Hui women with no formal schooling were confined to their households and farms and knew little about the outside world. They are less assimilated in health care institutions and practices, thus increasing their dependence on their husbands and families and furthering their disengagement from mainstream society.

While research has focused on individuals, some researchers believed that HL reflects the contextual demand on health care [15]. The structural barriers for individuals with low HL include the complex tasks, the limited access to the health care, and the inadequate preparedness of health care providers [15]. Existing payment structures may encourage health providers in village clinics and township health centers to emphasize medical services over health education, making health education a mere formality. Therefore, addressing the underlying structural factors may be especially important to improving women’s HRQoL by increasing HL. Cultural competence intervention for Hui minorities including minority recruitment into the health professions and provider education on cross-cultural issues may improve matters as well [45]. However, ethnic disparities in the association of HL with HRQoL may be underestimated because the Hui, representing 45% of population in the surveyed area, are not a “minority” in that region.

The converse is also true. Our study demonstrated that ethnic disparities in HRQoL were complex and varied by different levels of HL. Among women with low HL, ethnic disparities in pain/discomfort and anxiety/depression impairments were significantly larger than those among the high HL group. Low HL appears to aggravate ethnic disparities in HRQoL while high HL attenuates those disparities. The modification by HL suggests that improving HL may help alleviate ethnic disparities in HRQoL. If low HL is remediable through multifaceted and collaborative interventions and if the relationships are causal, this approach may be feasible and inexpensive [4]. HL can be improved not only by facilitating the acquisition of new knowledge and skills among these women but also by simplifying and standardizing health-related information through lower literacy burden of materials and health related tasks [4]. Nevertheless, it is essential to improve health system access among ethnic minorities with low HL; this would help to eliminate structural barriers to HL and to participation in the health care system.

The study has several limitations. First, the study was cross-sectional in design and hence the analysis did not permit causal inferences between HL and ethnic disparities in HRQoL. Second, HL is self-designed, which may influence its comparison with previous studies. However, the meaning and impacts of HL are complex, intricately linked to culture, language and facets of life that vary widely between different racial and socioeconomic groups [23]. Third, our study sample was comprised entirely of younger women (i.e. mothers of students), which could introduce a sampling bias. Further, ethnic disparities in HL and HRQoL may differ by gender. Future research should extend the focus to men in order to broaden and strengthen knowledge of ethnic minority health. Finally, results may not be generalizable to all poor minority areas in China because of the extensive socioeconomic and cultural disparities among these areas.

Despite these limitations, our study provides some valuable insights into the complex association between HL and ethnic disparities in HRQoL in a poor minority area of China.

## Conclusions

The HRQoL is very poor among rural women in the surveyed poor minority area. The most common impairments were pain/discomfort and anxiety/depression. HL is negatively associated with HRQoL across the entire sample; and the association may be modified by ethnicity, more pronounced among Hui minorities. Similarly, ethnic disparities in HRQoL may be modified by HL, larger in low HL group. Health interventions should be targeted to the vulnerable minority women to improve their HRQoL by addressing health literacy.

## Abbreviations

HRQoL: Health-related quality of life; HL: Health literacy; EQ-5D: European Quality of Life-5 Dimensions; WHO: World Health Organization; AMA: The American medical association; IOM: The institute of medicine; HLS-EU: European Health Literacy Project; PR: Prevalence ratio; CI: Confidence interval; CVIs: Content validity indexes; CAHLQ: The Chinese adult health literacy questionnaire; TOFHLA: The test of functional health literacy in adults.

## Competing interests

The authors declare that they have no competing interests.

## Authors’ contributions

CW designed the study, collected, analyzed and interpreted the data, and drafted the manuscript. HL and LL participated in its design and coordination. DX performed data analysis. RK and QM were involved in study concept and design, data interpretation and revision of manuscript. All authors read and approved the final manuscript.
